# Analyzing multicomponent receptive fields from neural responses to natural stimuli

**DOI:** 10.3109/0954898X.2011.566303

**Published:** 2011-07-22

**Authors:** Ryan Rowekamp, Tatyana O Sharpee

**Affiliations:** The Computational Neurobiology Laboratory, The Salk Institute for Biological Studies, La Jolla, CA 92037, USA and The Center for Theoretical Biological Physics, University of California, San Diego, La Jolla, CA, USA

**Keywords:** Information theory, natural scenes, single neuron computation, visual system

## Abstract

The challenge of building increasingly better models of neural responses to natural stimuli is to accurately estimate the multiple stimulus features that may jointly affect the neural spike probability. The selectivity for combinations of features is thought to be crucial for achieving classical properties of neural responses such as contrast invariance. The joint search for these multiple stimulus features is difficult because estimating spike probability as a multidimensional function of stimulus projections onto candidate relevant dimensions is subject to the curse of dimensionality. An attractive alternative is to search for relevant dimensions sequentially, as in projection pursuit regression. Here we demonstrate using analytic arguments and simulations of model cells that different types of sequential search strategies exhibit systematic biases when used with natural stimuli. Simulations show that joint optimization is feasible for up to three dimensions with current algorithms. When applied to the responses of V1 neurons to natural scenes, models based on three jointly optimized dimensions had better predictive power in a majority of cases compared to dimensions optimized sequentially, with different sequential methods yielding comparable results. Thus, although the curse of dimensionality remains, at least several relevant dimensions can be estimated by joint information maximization.

## Introduction

An essential element for achieving a quantitative understanding of sensory processing consists of characterizing the computational rules according to which the incoming stimuli are encoded within the sensory pathways. A useful and relatively laconic description of responses of a sensory neuron consists of specifying (1) the relevant stimulus features that affect its spike probability and (2) the nonlinear gain function that describes the dependence of the spike probability on the stimulus projections along these relevant dimensions. These features form the framework of the linear/nonlinear (LN) model (de Boer and Kuyper 1968; Shapley and Victor 1978; Meister and Berry 1999). The crucial simplification of this framework is that the number of relevant dimensions is assumed to be small.

Recent studies of neural responses to randomized stimuli have revealed that responses of many types of sensory neurons are modulated by more than one stimulus feature. In the visual system, at least two stimulus features were found to be relevant for responses of fly H1 motion sensitive neuron (Brenner et al. 2000a), retinal ganglion cells (Fairhall et al. 2006), the thalamic visual neurons (Sincich et al. 2009), and neurons in the primary visual cortex (V1) (Brenner et al. 2000a; Touryan et al. 2002, 2005; Rust et al. 2005; Chen et al. 2007; Rapela et al. 2010). Two-dimensional encoding was also observed for neurons in the somatosensory (Maravall et al. 2007) and auditory (Atencio et al. 2008) cortices. Finally, two or more relevant stimulus features can arise even as a result of basic nonlinear processes of spike generation as was demonstrated both in slice recordings from the brainstem nucleus magnocellularis (Slee et al. 2005) and in computations of model Hodgkin-Huxley neurons (Hong et al. 2007). Thus, multidimensional encoding appears to be quite ubiquitous and can arise both as a result of single neuron dynamics and computations at the circuit level.

Most of the studies demonstrating the presence of multidimensional encoding used randomized stimuli, such as white noise or correlated Gaussian noise. The next open question is to study multidimensional feature selectivity with natural stimuli. Such comparison is necessary because many aspects of neural responses exhibit adaptation to a host of statistical parameters of the stimulus distribution, including mean, variance (Fairhall et al. 2001; Maravall et al. 2007), or differences in the power spectra between noise and natural stimuli (Sharpee et al. 2006). Adaptation has often been observed on multiple time scales (Wark and Fairhall 2007; Lundstrom et al. 2008). It is desirable to study differences in the multicomponent feature selectivity between natural and randomized stimuli. However, we must first establish that the computational methods perform adequately given the constraints imposed by the statistics of natural scenes and available neurophysiological data. Several computational methods have been previously described in the literature for this purpose. These include finding relevant dimensions as those that maximize the amount of mutual information about the neural response (Sharpee et al. 2004a), maximization of other related objective functions (Paninski 2003; Sharpee 2007), and the projection pursuit regression (Rapela et al. 2006, 2010). Although information maximization has been shown analytically to estimate relevant dimensions with the smallest amount of variance in the limit of large datasets (Kouh and Sharpee 2009), the feasibility of a joint search of dimensions was demonstrated only for two relevant dimensions using model neurons with spatial (Sharpee et al. 2004a) or spatiotemporal (Sharpee et al. 2004b) features. Thus, it is not clear how well the performance of the joint search algorithm would fare for a larger number of dimensions. As the number of dimensions increases, the multidimensional probability distributions required for information maximization become increasingly noisy as the data are distributed across the growing number of histogram bins. This effect is known as the curse of dimensionality (Bellman 1961). A recent study by Rapela et al. (2010) proposed instead to search for dimensions sequentially. This is a very attractive possibility. However, the projection pursuit method relies on the assumption that the spike probability is a separable function of different relevant stimulus components. This assumption is unlikely to hold exactly for real neurons. In this article, we explore the relative advantages and disadvantages of the joint search and different types of sequential searches for the relevant stimulus dimensions with a focus on practical issues of reconstructing neural responses to natural stimuli within the framework of the LN model.

## Characterizing neural feature selectivity: Multidimensional linear-nonlinear model

Responses of sensory neurons can be modulated by a wide range of stimuli, from those that suppress their firing below the spontaneous rate to those that elicit near maximal firing rates. The classical LN model (de Boer and Kuyper 1968; Shapley and Victor 1978; Meister and Berry 1999) aims to account for all of these responses. Here, one assumes that the neural response is an arbitrary nonlinear function *g* of the degree of similarity (as measured by the projection value) between a given stimulus **s** and the relevant dimension *ê_1_*:

(1)
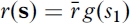


where 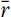 denotes the average spike rate across all stimuli, and 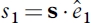 denotes the projection values onto the relevant dimension ê_1_. The nonlinear function *g* describes the modulation of the neuron's response relative to its mean firing rate. This function *g* can be an arbitrary, potentially highly nonlinear, function of the stimulus projections. Typical examples include sigmoid or threshold functions that are needed to describe such properties of neural responses as saturation and rectification. Beyond its first application to describe response properties of auditory neurons, the LN model has provided insights into the coding properties of neurons in many different sensory systems, including auditory (Theunissen et al. 2000, 2001; Sen et al. 2001; Hsu et al. 2004; Gill et al. 2006; Nagel and Doupe 2006, 2008; Woolley et al. 2006a,b), visual (Shapley and Victor 1978; Meister and Berry 1999; Chichilnisky 2001; Nykamp and Ringach 2002; Ringach et al. 2002; Ringach 2004; Fairhall et al. 2006), and recently olfactory (Geffen et al. 2009) neurons.

Recent studies have shown that extensions of this model allowing for the possibility of multiple relevant dimensions are necessary to better describe neural computations arising both from the dynamics of spike generation (Ag¨era y Arcas and Fairhall 2003; Ag¨era y Arcas et al. 2003; Hong et al. 2007) and circuit mechanisms, again in several sensory modalities including auditory (Atencio et al. 2008, 2009), somatosensory (Maravall et al. 2007), olfactory (Geffen et al. 2009), and visual (de Ruyter van Steveninck and Bialek 1988; Brenner et al. 2000a; Bialek and de Ruyter van Steveninck 2005; Rust et al. 2005; Fairhall et al. 2006; Chen et al. 2007; Sincich et al. 2009). In this extended multidimensional form, the spike probability is determined by an arbitrary nonlinear function *g* of *K* variables:

(2)
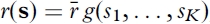


where 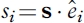 represent projection values of the stimulus s onto *K* relevant dimensions 
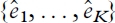
. It is also implicitly assumed that the number of relevant dimensions *K* is much smaller than the dimensionality *D* of the stimulus space. It should be noted that, for clarity and simplicity, this article uses the presence or absence of a single spike as the response of interest. Optimization procedures described below can be adapted for other types of responses, such as patterns of spikes across time or neural populations (Brenner et al. 2000b).

The reduction of dimensionality provided by the LN model makes analyzing neural responses to complex stimuli tractable, both in terms of its estimation from neural data and interpretation of results. Although each particular stimulus represents a point in a high-dimensional space, the model specifies that only a small number of dimensions are relevant for spike generation. At the same time, the LN model is quite versatile and can account for many types of neural responses. This is because relevant dimensions can represent arbitrary profiles in space, time, or other relevant variables, such as frequency for auditory neurons. Additional versatility is provided by the fact that the nonlinear gain function *g*(*s*_1_, …, *s_K_*) can take an arbitrary shape. The reduced dimensionality of the model makes it amenable for interpretation of results in terms of the computations performed. Profiles specified by the relevant dimensions represent the relevant stimulus features, and the nonlinear gain function describes how these features modulate the neural firing rate. Finally, the LN model also allows one to make predictions of the firing rate elicited by novel stimuli not used in the estimation of the model.

Here we focus on the problem of estimating multiple relevant dimensions from neural responses to natural stimuli. Although many types of natural stimuli have certain statistical properties in common, such as strong pairwise and higher-order correlations and other non-Gaussian properties (Ruderman and Bialek 1994; Schwartz and Simoncelli 2001; Simoncelli and Olshausen 2001; Lewicki 2002), the generative model for natural stimuli is not available. Therefore, the corresponding statistical methods that we will test do not rely on any specific assumption about stimulus statistics. In testing the methods for estimating the multidimensional LN model, we will compare their performance with natural and more randomized stimuli, such as uncorrelated Gaussian noise.

### Maximally informative dimensions

Given a set of stimuli and responses, the estimation of the LN model consists of two parts. The first task is to find a subspace that determines the activity of the neuron. The second task is to estimate the nonlinear function *g*. The first task is much more computationally difficult than the second task. This is because given a set of dimensions, *g* can be determined empirically using

(3)
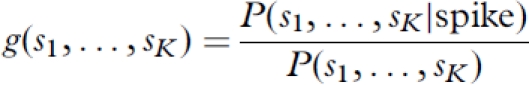


We note that the problem is formulated in terms of the relevant subspace (Sharpee et al. 2004a): any non-degenerate linear combination of vectors 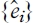 will span the same subspace and provide an equivalent description of the neural responses.

A number of methods and objective functions can be used to fit the LN model to the data. Early approaches for fitting one-dimensional LN models (Hunter and Korenberg 1986) relied on iterative updating between the estimates of dimensions and the corresponding gain functions. However, this method only works with monotonic gain functions that can be inverted, and thus cannot be applied to find multiple relevant dimensions. A complementary approach known as the spike-triggered covariance method that is suitable for multidimensional LN models consists of diagonalizing the difference between second-order matrices of all presented stimuli and those that elicited a spike (de Ruyter van Steveninck and Bialek 1988; Bialek and de Ruyter van Steveninck 2005; Schwartz et al. 2006). The corresponding eigenvectors yield the relevant dimensions associated with a spike, and no separate optimization of the nonlinear gain function is required. However, the spike-triggered covariance method, as well as its information-theoretic generalizations (Pillow and Simoncelli 2006) that achieve better convergence by utilizing the sensitivity of the mutual information to changes in both the mean and covariance, are unbiased only with Gaussian stimuli (Bussgang 1952; Paninski 2003; Bialek and de Ruyter van Steveninck 2005). This suggests that fitting of the multidimensional LN model to data from non-Gaussian stimuli requires optimization of both the estimates of relevant dimensions and the corresponding multidimensional nonlinear gain function.

Fortunately, optimization of the nonlinear gain function can be done automatically by incorporating it into the definition of the objective function that evaluates the quality of fit. This is possible because Equation (3) provides a closed form expression for the optimal gain function for a given set of dimensions. Therefore, instead of explicitly computing the predicted firing rate for all presented stimuli and comparing them to the measured values, it is possible to write down the corresponding measure of the quality of fit that will depend explicitly only on the dimensions themselves. For example, one might wish to find the LN model that yields firing rate predictions that best match experimentally measured values in least squares terms. The corresponding objective function for evaluating a candidate set of relevant dimensions {**v**_*i*_} is given by the Rényi divergence of order 2 between probability distribution of stimuli *P*(*x*_1_, …, *x_K_*) along *K* dimensions under consideration and the analogous probability distribution *P*(*x*_1_, …, *x_K_*|spike) that is computed across the subset of stimuli that elicited spikes:

(4)
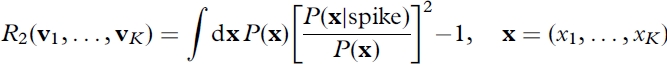


where the probability distribution *P*(**x**) and *P*(**x**|spike) are defined as

(1)
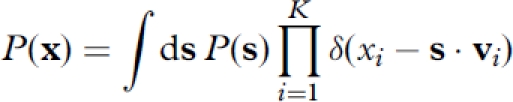


and

(1)
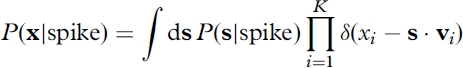


where δ(*x*) is the Dirac delta function and *x_i_* = **S · V_i_**. These distributions can be determined empirically by binning the projection values into normalized histograms, which would approximate *g* as a piecewise constant function, or by fitting them using parametric distributions, such as Gaussian (Pillow and Simoncelli 2006) or exponential (Paninski 2004). Although with sufficient data any continuous function can be approximated to a desired accuracy with a piecewise constant function, practical limitations may prevent this (see Section ‘Discussion’ for further detials).

Rényi divergences of other orders can also be used as an objective function. These objective functions explicitly depend only on the relevant dimensions (**V**_1_, …, **V**_*K*_). However, the ratio of probability distributions that appears in their expression represents the nonlinear gain function (compare Equations (3) and (4)), which is thus implicitly taken into account. Although optimization of Rényi divergence of any order will yield correct relevant dimensions in the limit of infinite data, these objective functions yield different estimation variance in the case of finite data. Among different orders of Rényi divergences, the Kullback-Leibler divergence, which is also a Rényi divergence of the first order, not only produces dimensions with the smallest variance compared to other Rényi divergences, but also saturates the Cramér-Rao bound, and thus achieves the smallest variance possible for any unbiased estimator (Kouh and Sharpee 2009). Similar to how the Rényi divergence of order 2 corresponds to minimizing the least squares difference between measured and predicted firing rates, maximization of the Kullback-Leibler divergence maximizes the amount of information captured by a given set of dimensions:

(7)
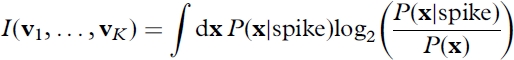


The amount of information *I*(**V**_1_, …, **V**_*K*_) accounted for by a given set of *N* dimensions cannot exceed the mutual information between spikes and unreduced stimuli:

(8)
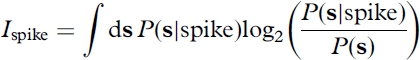


According to the data processing inequality, reducing stimuli to a set of projections will decrease the information unless all of the discarded dimensions do not influence the spike probability. This suggests that the number of relevant dimensions can be determined by iteratively increasing the number of vectors being optimized until the information explained approaches *I*_spike_. Once this is achieved, no additional information remains to be accounted for and thus all of the relevant dimensions have been found. From a practical standpoint, the information per spike *I*_spike_ can be estimated by observing variations in neural responses to repeated stimuli, calculating the information for various lengths of data, and extrapolating to infinite data (Strong et al. 1998; Brenner et al. 2000b). This circumvents the problem of exploring the entire stimulus space in Equation (8).

## Comparison of sequential and joint optimization of relevant stimulus dimensions

The central question that we would like to address in this article is whether the relevant stimulus dimensions can be found one-by-one through a series of one-dimensional optimizations or whether a truly multidimensional, joint optimization of the relevant dimensions is required. One way to perform a sequential search is to optimize information along a single dimension,

(9)
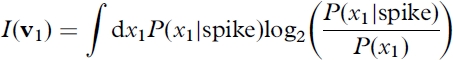


to find the first maximally informative dimension (MID-1), and then continue to optimize this function by restricting the search to all dimensions that are orthogonal to the first, and all of the subsequently found dimensions. This procedure represents a very attractive possibility, and it can be carried out not only by maximizing information, but also by maximizing the percentage of variance explained using a one-dimensional version of Equation (4). Optimization of any one-dimensional objective function only requires the sampling of one-dimensional input/output functions, which have lower requirements for the recording size and computational time compared to computations of multidimensional gain functions, as in Equation (7).

### Analysis of systematic bias of sequential information maximization

To validate any sequential optimization, we need to verify that (i) the estimates of dimensions that are computed first are not biased by the presence of other relevant dimensions and (ii) the estimates of subsequently computed dimensions are not biased by restricting the search to the subspace orthogonal to the previously found dimensions. The two effects are related. However, we demonstrate below that the presence of stimulus correlations has a stronger effect on the estimation of subsequent dimensions, mainly through stimulus correlations with the previously found dimensions. These issues do not represent a problem during a joint optimization, because it provides an opportunity to adjust all relevant dimensions and interactions between them.

To address these questions analytically, let us consider the case where only two stimulus dimensions are relevant. The first question is whether the estimate of the first dimension will be biased because the second dimension was ignored during the one-dimensional search. Let *ê*_1_ be the maximally informative linear combination of the relevant dimensions and *e*_2_ be the component of the relevant subspace that is orthogonal to *ê*_1_. The gradient of information when evaluated along *ê*_1_ is given by (Sharpee et al. 2004a):

(10)
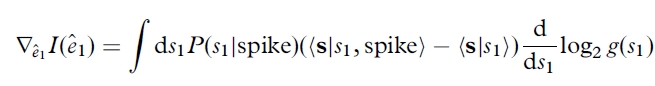


If the gradient 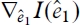 is non-zero, then the estimated relevant dimension will be pulled out of the relevant subspace, which leads to a biased reconstruction. It is convenient to expand expression (10) within the relevant subspace using the following identities: 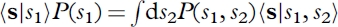 and 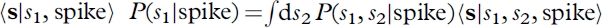. Noting that 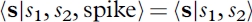 because *s*_1_ and *s*_2_ are sufficient statistical variables (i.e. they contain all of the information between the stimulus and spikes), Equation (10) can be written as

(11)
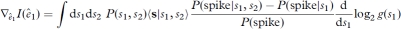


This last expression illustrates that, if the conditional average 〈**s**|*s*_1_, *s*_2_〉 of stimuli with projections *s*_1_ and *s*_2_ onto the two relevant dimensions has components outside of the relevant plane, the gradient of information may also have such non-zero components. This average can be expressed as 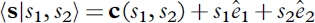 with **c** being the component orthogonal to the relevant subspace. In the case of uncorrelated Gaussian stimuli, **c** is a constant vector. Its contribution integrates out to zero in Equation (11) because

(12)
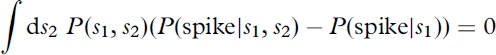


Therefore, in the case of uncorrelated stimuli, the gradient of information along the maximally informative vector within the subspace will have no components outside the relevant subspace. Thus, the maximally informative vector within the subspace will also be the maximally informative vector across the entire stimulus space. This argument can be extended to cases where c is either independent of *s_2_* or varies linearly with *s_2_*, which is the case for correlated Gaussian stimuli. Components that are independent of *s_2_* integrate to zero because of Equation (12) and the components that increase linearly with *s_2_* integrate to zero because they are proportional to the gradient of the information along *ê*_2_ which is zero according to the definition of *ê*_1_ as the maximally informative dimension within the relevant subspace. Thus, if stimuli are Gaussian (with or without correlations), then the first dimension obtained through one-dimensional optimization does not need to be updated once other relevant dimensions are estimated. The same could still be true in the case of correlated non-Gaussian stimuli, but not generically. For example, one condition that is sufficient to ensure that the first dimension does not need to be updated using the joint search is if the conditional spike-triggered average 〈**s**|*s*_1_, spike〉 – 〈**s**|*s*_1_〉 is zero for all values of *s_1_* (cf. Equation (10)). One way in which this could happen is if (i) the nonlinear gain function is symmetric with respect to *s_2_* and –*s_2_* and at the same time, (ii) the conditional stimulus probability distribution *P*(*s*_2_|*s*_1_) has a mean of zero. These two conditions (i) and (ii) should hold for any value of *s_1_*. Thus, in the case of natural stimuli, which are strongly non-Gaussian (Ruderman and Bialek 1994; Schwartz and Simoncelli 2001; Simoncelli and Olshausen 2001), it appears likely that previously found dimensions would need to be updated.

Similar arguments can be used to analyze the bias in the subsequently found dimensions. Our goal now is to verify that the gradient of one-dimensional information 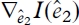, which can be computed analogously to Equation (11):

(13)
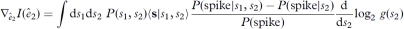


has no components along vectors other than *ê_1_* (components along *ê_1_* do not represent a problem, because they are removed by restricting the search to the subspace orthogonal to *ê_1_*). In the case of uncorrelated stimuli, 〈**s**|*s*_1_, *s*_2_〉 is a constant vector. Taking into account that

(14)
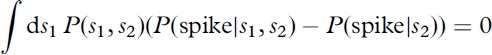


we find that the gradient 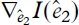 will have no components along dimensions other than *ê_1_* and *ê_2_*. Thus, the sequential optimization is valid for any type of neural gain function if stimuli are uncorrelated.

If stimuli are correlated, then 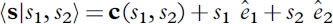, where the vector **c**(*s*_1_, *s*_2_) has components along irrelevant dimensions whose magnitude may increase linearly with *s_1_* (higher-order terms are also possible for correlated non-Gaussian stimuli). In the general case, the expression

(15)
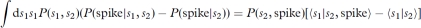


is non-zero; it is proportional to the component of the gradient of along *ê*_1_. Thus, the gradient 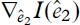 may have components along irrelevant dimensions. For example, a one-dimensional search for the second informative dimension may yield a dimension that is strongly correlated with the first MID (although orthogonal to it), but provides little extra information in addition to *ê_1_* when evaluated using the joint information.

In summary, the sequential search for relevant dimensions from neural responses to natural stimuli faces two problems. First, although estimation of the first dimension is not affected by stimulus correlations of the second order, it may be affected by stimulus correlations of higher orders (which represent non-Gaussian effects previously demonstrated for natural stimuli). Second, the sequential estimation of secondary dimensions may be affected by both second-order and higher-order correlations. Below we will examine the strength of these effects by comparing results of joint and sequential optimization for model cells with two and three relevant stimulus dimensions.

For the joint search, these effects are not of concern. When the number of optimized dimensions is equal to the number of relevant dimensions, the information maximum is guaranteed to occur at the relevant dimensions by the data-processing inequality. Furthermore, the gradient of information is computed with respect to a single dimension but which takes into account other relevant dimensions:

(16)
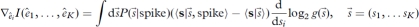


is zero for any linear combinations of the relevant dimensions.

### Analysis of systematic bias of projection pursuit regression

Our discussion so far has been focused on comparing sequential and joint optimizations in the context of information maximization. We chose information maximization among other Rényi divergences because its estimation variance is the lowest possible for joint optimization and because it represents a way to perform maximum likelihood fitting that is adapted to the structure of the LN model (Kouh and Sharpee 2009). However, in the case of sequential optimization, other strategies are possible. The classic projection pursuit strategy is one of the most widely used (Friedman and Stuetzle 1981), and it was recently adapted to analyze multi-component neural feature selectivity (Rapela et al. 2006, 2010). Projection pursuit regression (PPR) models approximate the neural response function as a sum of one-dimensional functions of projections onto individual dimensions. This model can be fitted using least squares regression. Here, each subsequent dimension is computed using least squares regression to describe the residual between the neural firing rate and its predictions based on all of the previously found dimensions.

To analytically investigate under what circumstances the projection pursuit regression has no systematic biases, we first consider the case of a one-dimensional (1D) LN model, and then generalize the argument to the multidimensional case. In the case of a 1D LN model, previous work has demonstrated that a mismatch between the model and neural nonlinear gain functions does not generate a systematic bias in the estimates of the relevant dimension as long as stimuli are Gaussian, with or without correlations (Ringach et al. 2002; Sharpee et al. 2004a). Those arguments were made in regards to the method of spike-triggered average by demonstrating that the estimation of the relevant dimensions was not affected by the linear approximation of the nonlinear gain function. Here we provide an alternative derivation that is tailored to the PPR method and can be generalized to multidimensional LN models.

The relevant dimension is computed by minimizing the least square difference between the measured and predicted firing rates :

(17)
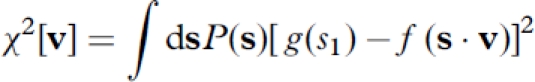


Here, we denote as *f* the fitted nonlinear gain function from PPR, which might differ from the neural gain function *g*(*s*_1_) from Equation (1). If neural spikes are indeed based on one relevant dimension *e*_1_ and the functional form *f* makes possible for it to match the nonlinear gain function *g*(*s*_1_), then χ^2^ difference will reach its minimal value of zero for **v** = ê_1_. Let us examine the magnitude of the χ^2^ gradient:

(18)
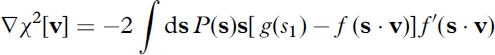


When evaluated at **v** = ê_1_

(19)
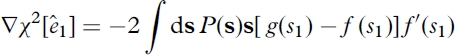


so that the gradient is indeed zero whenever *f=g*. Furthermore, it can be shown that the gradient will also be zero if stimuli are Gaussian (with or without correlations). This can be demonstrated by carrying out the averaging in Equation (19) with respect to all possible input components, except for *s_1_*:

(20)
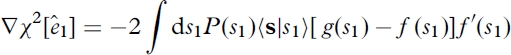


We recall that in the case of Gaussian stimuli, 〈**s**|*s*_1_〉 = **c**_1_ *s*_1_, where **c**_1_ is a constant vector (the mean stimulus was set to zero because PPR relates stimulus variations to the firing rate variations). Therefore, the magnitude of the gradient in all directions is proportional to the magnitude of the gradient along *ê_1_*. In other words, when the appropriate scale for the relevant dimension is found, the gradient will be zero in all directions. Thus, in the case of Gaussian stimuli (with or without correlations), the relevant dimensions can be computed with zero systematic bias even in the presence of a mismatch between the neural *g* and model *f* gain functions. With non-Gaussian stimuli, PPR will also provide a good estimate of the relevant dimension in the situation where the spike probability is described by a 1D LN model. This is because any continuous function can be uniformly approximated to an arbitrary degree of precision with a set of polynomials (Rapela et al. 2010). The situation is different, however, when neural responses are affected by multiple stimulus components.

When a multidimensional LN model is necessary to describe the neural responses, we can again look for dimensions that minimize the least square difference (for illustration purposes we consider a 2D case):

(21)
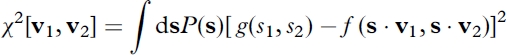


The gradient of χ^2^ with respect to either the first (and analogously the second) dimension when evaluated with **v**_1_ = ê_1_ and **v**_2_ = ê_2_ is given by

(22)
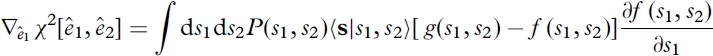


As before, for Gaussian stimuli the components of the conditional average 〈**S**|*s*_1_, *s*_2_〉 are linearly dependent on *s*_1_ and *s*_2_. In this case therefore, when the magnitude of the relevant dimensions reaches such values that the components of the gradient along *ê_1_* and *ê_2_* are zero, then all other components of the gradient will also be zero. With non-Gaussian stimuli, the gradient of χ^2^ will have no components along irrelevant dimensions only when *g*(*s*_1_, *s*_2_) can be well approximated by the model neural gain function *f*(*s*_1_, *s*_2_). Unfortunately, this is in many cases not possible, because the essential feature of PPR is that it considers nonlinear gain functions that are sums of nonlinear functions of different components, such as *f*_1_(*s*_1_) + *f*_2_(*s*_2_), whereas non-separable nonlinear gain functions are prominent in neural LN models (Rust et al. 2005; Fairhall et al. 2006; Chen et al. 2007; Atencio et al. 2008, 2009). Furthermore, one can expect systematic biases even in the estimate of a single PPR dimension because the one-dimensional gain function cannot fully account for the multidimensional nonlinear gain function. In summary, analytical considerations suggest that one should expect to find similar systematic biases in the relevant dimensions computed from neural responses to natural stimuli with either sequential information maximization or projection pursuit regression.

### Numerical algorithms

In what follows, we compared relevant dimensions that were reconstructed using either sequential or joint optimization of information or PPR. The latter has been recently described by Rapela et al. (2010) and is publicly available at http://vpl.usc.edu/projects/ePPR/. Therefore, we focus here on the differences between the algorithms for joint and sequential optimization of information. For the first dimension, sequential and joint optimization are equivalent. The sequential search (as well as the search for the first dimension) optimizes information given by Equation (9) with the gradient computed according to Equation (10). Components of the gradient along the first and subsequent dimensions are removed. This forces the optimization procedure to search within the space orthogonal to all previously found dimensions. As pointed out above, this has the advantage of allowing us to avoid calculating multidimensional probability distributions, which become increasingly noisy as the data are distributed across the growing number of histogram bins required for multiple dimensions. The joint optimization maximizes full information of Equation (7) using the gradient given by Equation (16). For most iterations, the gradient is taken with respect to the new dimension, but every 100th iteration of the algorithm updates the previously found dimensions. Optimizing previous dimensions is necessary to remove biases caused by correlations between the relevant dimensions and other stimulus dimensions. We observed that in few cases this changed the previously found dimensions by a substantial amount.

Except for the difference in optimization functions and their gradients, the numerical algorithms for performing joint and sequential information maximization were identical. The optimization algorithm was based on the combination of simulated annealing and gradient ascent (Press et al. 1992; Sharpee et al. 2004a, 2006). Simulated annealing allows the algorithm to escape local maxima by choosing trial dimensions with lower information value with the probability ∝ exp (Δ*I/T*), where Δ*I* is the difference in information values at the current and a tested point in the stimulus space, and parameter *T* (effective temperature) controls the magnitude of decreases in information values that are accepted often (increase in information are always chosen as new optimization points). The effective temperature *T* is gradually decreased (by a factor 0.95) with each iteration until the algorithm converges to a local maximum. When this happens, the temperature is increased and the optimal point is perturbed by a large step, allowing it to follow the gradient to another, possibly better maximum. The optimization continued for fixed number of line optimizations (which is one of the adjustable parameters within the publicly available version at http://cnl-t.salk.edu/Code/). In the analysis of the simulated model cells and recordings from the visual cortex, each new dimension was optimized for 1200 iterations, with the number of bins stepping from six to eleven every 200 iterations.

To mimic as closely as possible the analysis steps involved in working with neurophysiological data, each dataset was analyzed four times by omitting a different 1/4 of the data. This resulted in four jackknife estimates of the relevant dimensions (Efron and Tibshirani 1998), and in what follows we report results as averages across these four estimates. The starting point in the calculation of the first dimension was computed as the spike-triggered average across the subset of stimuli that did not overlap with other jackknifes. The search for subsequent dimensions used one of the stimulus frames as the starting point. This frame was different for each new dimension and each jackknife estimates. In this way, the variability across jackknifes reflects the contributions from variability across the stimulus subsets and the effects of local maxima and starting conditions during the non-convex information maximization.

### Subspace projection for evaluating reconstructions of model dimensions

One can evaluate the quality of the reconstructed dimensions by comparing the similarity of the subspace they define to that defined by the relevant dimensions of the model. If the subspaces are the same, then they simply represent alternative coordinate systems that provide equivalent descriptions of the neural firing. One way to measure this similarity is by measuring the intersection volume between unit cubes of the two subspaces. If 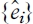 and 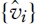 are orthonormal sets of unit vectors defining the model and reconstructed subspaces, respectively, then the Jacobian of the transformation from model dimensions to the projections of reconstructed dimensions onto the model subspace is given by a matrix 
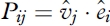
. The determinant of the Jacobian matrix represents a change in volume associated with this transformation (Arfken and Weber 1970). Thus, |det(*P*)| represents the volume of the reconstructed unit cube that remains when projected onto the model subspace. This expression can be generalized to the case of arbitrary sets of basis vector rather than sets of orthonormal unit vectors by dividing it by 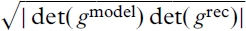, where matrices 
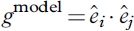
 and 
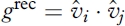
 are the metric tensors (also known as Gram matrices) of the model and reconstructed subspaces, respectively. This is because the volume spanned by a set of dimensions equals a square root of the metric tensor determinant (Dodson and Poston 1991; Gradstein and Ryzhik 2000). Thus, in the general case the projected volume is given by

(23)
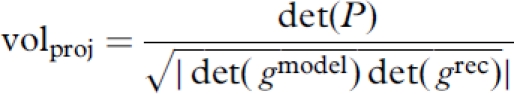


Finally, we convert the projected volume into a linear measure by taking the i>Cth root (where *K* is the number of relevant dimensions):

(24)
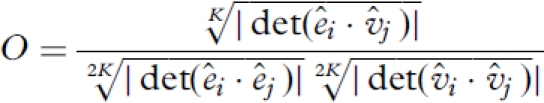


The linear subspace projection *O* defined in Equation (24) ranges from zero, indicating that the subspaces have no relation to each other, to one, indicating that the subspaces are identical. For one dimension, it is simply the scalar product. For *K* > 1, as mentioned above, it represents the linear dimension of the hypercube whose volume represents the overlap between the model and reconstructed dimensions. Quantifying the amount of overlap between the model and reconstructed subspace in terms of linear dimensions (i.e., taking the *K*th root) ensures that the values do not become exponentially small solely due to the increased dimensionality of the subspace. For example, if each of the three hypothetical relevant dimensions are reconstructed with projection values of 0.8, the linear subspace projections is O = 0.8, whereas the volume of the overlap is only ≈0.5. Unlike the principal angles (Rapela et al. 2010), the subspace projection measure (Equation (24)) has the advantage of being rotationally invariant, and is not affected if the reconstructed dimensions are not orthogonal or cluster around one particular model dimension.

### Simulations on model visual cells with spatiotemporal dimensions

**Figure 1:**
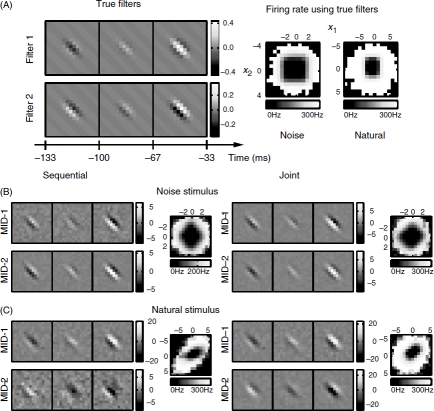
Joint optimization is required in the case of natural stimuli. (A) Spatiotemporal filters of the model neuron and associated nonlinear gain functions. Nonlinear gain functions were calculated from model responses to noise and natural stimuli. Axes of nonlinear gain functions are shown in units of standard deviation of the corresponding relevant dimensions. (B) Reconstruction in the case of noise stimuli. Optimization of one-dimensional information is sufficient to reconstruct the relevant dimensions of the model (subspace project O = 0.83±0.15). Joint optimization of two-dimensional information did not further improve the result (O= 0.8±0.2). (C) Natural stimulus. Joint optimization of relevant dimensions by maximizing the two-dimensional information markedly improves the subspace projection between model and reconstructed dimensions from 0.60 ±0.04 to 0.875 ±0.008. Note that the rotated nonlinear gain function in the relevant plane provides an equivalent description the system. Color-scale for each of the reconstructed filters represents signal-to-noise ratio.

Our first neural model was constructed according to an LN model based on two relevant dimensions. These dimensions described spatiotemporal filters at three time points and on a spatial grid of 16 × 16 pixels (Figure 1A). At each moment in time, both filters consisted of spatial Gabor filters with identical orientation and spatial frequency but with orthogonal phases (DeAngelis et al. 1993). The time dependence described by the filters was chosen to yield sensitivity to the onset of the preferred spatial feature. A spike was produced according to a logical OR, that is, if the absolute value of the stimulus components along either dimension 1 or dimension 2 exceeded a threshold value *θ* in the presence of Gaussian noise with variance *σ^2^*. The corresponding nonlinear gain function obtained after averaging with respect to noise is also shown in Figure 1(A). In order to test analytical predictions described above for the validity of sequential optimization for Gaussian and non-Gaussian stimuli, we generated responses of this model cell to two ensembles: white noise stimuli (Gaussian) and natural stimuli. Both stimulus ensembles had the same number of frames (∼50 000). The average spike rate was 47 and 42 Hz for noise and natural stimulus ensembles, respectively (assuming that the frame rate of 33 Hz).

In agreement with theoretical arguments based on properties of the gradient (Equation (11)), we found that in the case of noise stimuli, the two relevant dimensions could be correctly reconstructed with a sequential search (Figure 1B). The subspace projection value was *O* = 0.83 ±0.15 (mean across jackknifes±SEM). The joint optimization of dimensions did not improve results further (O = 0.8 ±0.2, cf. Figure 1C). In addition to the subspace projection, one can also evaluate the performance of the reconstructed dimensions by computing the log-likelihood of the corresponding LN model on a novel dataset. In the limit of small spike probabilities within each bin of the spike train (which is also the assumption underlying the fitting of LN models), the log-likelihood is proportional to the mutual information (Kouh and Sharpee 2009). The relevant dimensions reconstructed using the sequential and joint search accounted for 78.6 ±0.6% and 83.8 ± 0.6% of the model information, respectively. Therefore, in the case of neural responses to noise inputs, sequential and joint optimization of relevant dimensions produced comparable results (the small difference in predictive power is likely due to small non-Gaussian effects introduced during the discretization of intensity levels). In summary, when analyzing the neural responses to Gaussian noise stimuli, there is no need to perform the increasingly onerous task of calculating multidimensional information (Equation (7)) for an increasing number of dimensions, and instead the relevant dimensions can be found sequentially.

In contrast to the responses to noise stimuli, and again in agreement with the theoretical analysis, in the case of natural stimuli sequential optimization of one-dimensional information was not sufficient to correctly reconstruct relevant dimensions. The reconstruction results using sequential optimization (Figure 1B) had a subspace projection with the model of 0.60 ± 0.04. Further, joint optimization yielded a significant improvement to 0.875 ± 0.008 in terms of subspace projection value, as well as the improved visual match between the reconstructed Gabor features to the model ones (Figure 1C). The percent information explained on a novel dataset was 63 ±3% for the sequential search and 90 ±4% for the joint search. These simulations illustrate that the non-Gaussian correlations in natural stimuli are strong enough to qualitatively and quantitatively alter results of the sequential optimization away from the true (model) relevant dimensions. These deviations can be corrected by a joint optimization (Figure 1C). Thus, the numerical simulations support the theoretical analysis in demonstrating that the joint optimization of relevant dimensions is required for analyzing multicomponent feature selectivity based on neural responses to natural stimuli.

### Comparison with projection pursuit regression

**Figure 2:**
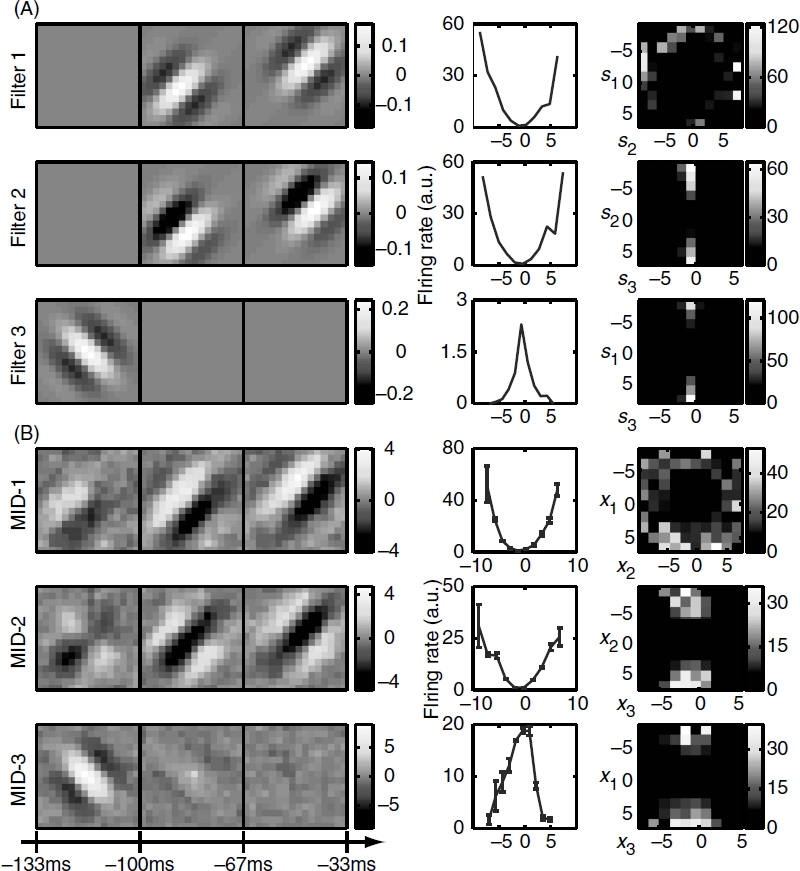
Reconstruction of a model neuron with three relevant dimensions using joint search for three maximally informative dimensions (MID). (A) The three spatiotemporal filters of the model cell (left, each filter is normalized to length one, as in Figure 1A) and associated nonlinear gain functions with respect to each of the relevant stimulus component considered individually (middle) or in pairs (right). Nonlinearities are calculated from model responses to natural stimuli. (B) Results of the reconstruction from responses to natural stimuli (the number of stimulus frames was ∼50 000). Optimization of joint information between spikes and three dimensions is able to recover all three dimensions of the model. The subspace projection between the model and reconstructed dimensions is 0 = 0.829 ±0.005, cf. Equation (24). Notations are as in Figure 1.

To further compare the performance of joint optimization with a sequential method, in this case projection pursuit regression, we used a model cell with three relevant dimensions, shown in Figure 2. This model cell was similar to the two-dimensional model considered above but contained a simplified version of a divisive gain control (Heeger 1992). The mean response of the cell was given by

(25)
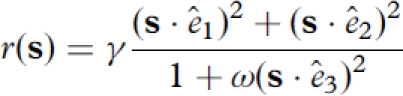


where the parameter γ controls the mean response and its variance (the response probability is a Poisson model), ο controls the relative strength of the suppressive dimension, and *ê*_1_, *ê*_2_, and *ê*_3_ represent three relevant spatiotemporal dimensions. Such a model cell provides a good testing ground for both joint information maximization and projection pursuit methods for the following three reasons. First, it allows us to test whether joint optimization of three dimensions can be done reliably during information maximization. Second, the nonlinear gain function in Equation (25) is not fully separable, and thus can serve as an illustration of how projection pursuit methods can work with more realistic nonlinearities that are likely to not be fully separable. Finally, this model cell was recently used to study multidimensional feature selectivity (Rapela et al. 2010). To ensure continuity with previous studies, we chose the same values for parameters in the gain function (Equation (25)) and modeled the spatiotemporal filters as close as possible to that study. We set γ such that (*r*(**s**)) was equal to 0.56 and ο such that 〈1 +ο(**s**·ê_3_)^2^〉 was equal to 4.26. We simulated responses of this model neuron to natural stimulus ensembles of two lengths, 20 000 frames and 49152 frames. The shorter stimulus ensemble had the same length as in Rapela et al. (2010). The longer stimulus ensemble is toward the upper limit of what can be reliably obtained in physiological recordings at the present time.

Results of the joint search for three relevant features with information maximization are shown in Figure 2. All of the three relevant dimensions could be reconstructed well using joint information maximization. The subspace projection (Equation (24)) was *O =* 0.829 ± 0.005 for the longer stimulus and *O =* 0.65 ± 0.08 for the shorter stimulus. The percent information explained on a novel dataset was 89 ± 6% for dimensions found using the longer stimulus and 83 ± 6% for dimensions found using the shorter stimulus. Both the excitatory dimensions *ê*_1_ and *ê*_2_, and the so-called suppressive dimension *ê*_3_ can be recovered by joint information maximization. (This terminology derives from the effects of these dimensions on the neural spike probability (Rust et al. 2005; Schwartz et al. 2006; Chen et al. 2007)). The nonlinear gain functions computed with respect to the reconstructed dimensions also yielded dependencies that were in agreement with the model, taking into account that the reconstructed dimensions represent linear combinations of model dimensions. This demonstrates that the joint information maximization can estimate up to three relevant dimensions. This is no small feat, because a three-dimensional nonlinear gain function formally requires its estimation at 1331 points (the maximal number of bins used at final stages of optimization was 11 along each dimensions), in addition to the spatiotemporal grid points that describe the filters themselves.

**Figure 3:**
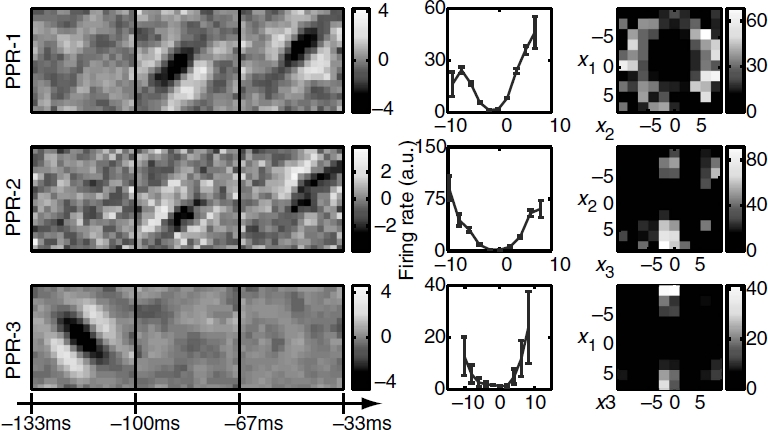
Reconstruction of a model neuron with three relevant dimensions using projection pursuit regression (PPR). The three relevant dimensions of this model neuron are shown in Figure 2 and the nonlinear gain function is given by Equation (25). The reconstruction was done on the longer natural stimulus ensemble (∼50 000 frames). The subspace projection between the model and reconstructed dimensions is 0 = 0.59 ±0.11, smaller than that obtained with the joint search for relevant dimensions using information maximization. Notations are as in Figure 1.

Analysis of the same sequence of neural responses using projection pursuit regression is shown in Figure 3. This analysis was done using the algorithm of Rapela et al. (2010), which is publicly available at http://vpl.usc.edu/projects/ePPRV. Reconstructions obtained by projection pursuit had subspace projection values of *O* = 0.59 ±0.11 and *O*=0.55 ±0.05 for the longer and shorter stimuli, respectively. The percent information explained was 81 ± 6% for dimensions found using the longer stimulus and 72 ±5% for dimensions found using the shorter stimulus. These values were lower than the values obtained using joint information maximization. Thus, in the case of natural stimuli, the estimation of relevant dimensions is strongly affected by the separable assumption made in the PPR method for the form of the nonlinear gain function. We also note that when accounting for the neural responses to natural stimuli, relatively small differences in the mutual information can indicate a large mismatch between the model and relevant dimensions (Sharpee et al. 2004a). This is because even a random dimension will have some component along the relevant dimensions and with the help of stimulus correlations can account for a noticeable portion of the overall information. Indeed, we observe that differences in the subspace projection were more pronounced between different methods than those in the information explained, although both measures are consistent with the better performance of the joint information maximization.

## Convergence properties of joint and sequential optimization

**Figure 4:**
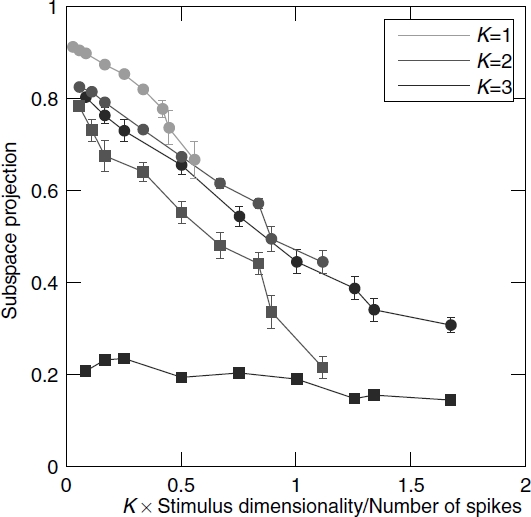
Convergence of the joint and sequential information maximization with increasing number of spikes. Subspace projection, Equation (24), between the model and reconstructed dimensions (*y*-axis) is plotted as a function of the ratio of stimulus dimensionality (*D*) times the number of relevant dimensions *(K)* to the number of spikes (*x*-axis). Small *x*-values correspond to good sampling (high signal-to-noise ratio). Results for model cells with *K* equal to 1, 2, and 3 are shown in green, red, and blue, respectively. Results obtained with joint information maximization (circles) are always better than those obtained with sequential information maximization (squares). By construction, they are identical for 1D model cell. In the case of 3D model cell, sequential optimization did not converge to the true subspace with increasing number of spikes. Note that the number of spikes needed to achieve the same quality of reconstruction increases linearly with the number *K* of reconstructed dimensions.

Simulations presented above demonstrated that, given a sufficiently long recording, up to three relevant dimensions, each consisting of hundreds of points can be estimated from neural responses to natural stimuli. In this section we examine how fast the reconstruction results deteriorate as the number of spikes decreases (Figure 4). The three-dimensional model cell was the same the one shown in Figure 2, with the nonlinear gain function described by Equation (25). The one-and two-dimensional model cells were similar, using the first one and two model filters, respectively. The two-dimensional model cell was obtained by setting ο = 0. The nonlinear gain function for a one-dimensional model cell was given by *r*(**s**) = *γ* (**S** · ê_1_)^2^. To reduce the number of spikes, we systematically lowered the average firing rate. Because the number of spikes was determined using a Poisson generator, this is equivalent to using fewer repetitions of the stimulus. For each model cell and firing rate, we used eight different simulations with different random seeds.

For joint optimization, we found that reasonable reconstructions of either two or three dimensions (defined as subspace projections values >0.5) can be obtained as long as the number of spikes is greater than the number of parameters needed to define the dimensions. The estimation curve for a single dimension was significantly better than those for two and three dimensions. An encouraging sign, however, is that performance at estimating two or three dimensions is comparable, and no drastic drop off is observed when expanding the number of relevant dimensions of LN model from two to three. Indeed, the convergence curves for the joint information maximization largely overlap when plotted as a function of *KD/N*_spikes_. This means that roughly three times as many spikes are needed to reconstruct three dimensions as are sufficient to reach the same degree of overlap when reconstructing a single dimension. Sequential optimization had some success with the two-dimensional model cells, although joint optimization performed better. However, for the three-dimensional cell, sequential optimization consistently performed poorly even in cases where a large number of spikes was available.

## Application to cells from the primary visual cortex (V1)

Having tested the algorithm on model neurons, we now use joint information maximization to reconstruct three spatiotemporal dimensions for neurons in the primary visual cortex (V1). Our results are not intended as a comprehensive analysis of multidimensional feature selectivity in V1, but rather as a proof-of-principle demonstration whether physiologically meaningful (and plausible) dimensions can be computed from neural responses to natural stimuli.

**Figure 5:**
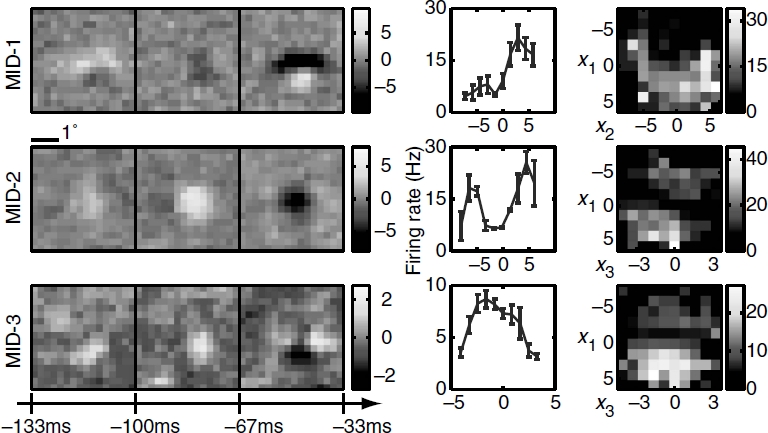
Reconstruction of an example V1 simple cell, (left) Two excitatory spatiotemporal filters (MID-1 and MID-2) and one inhibitory filter (MID-3), (right) The associated one-and two-dimensional non-linear firing rate functions are consistent with the properties of simple cells and nonlinear gain control model. Neuron 761 — 1.

Three relevant spatiotemporal features and the corresponding nonlinearities for an example simple cell are shown in Figure 5. All three relevant dimensions could be reliably estimated (signal-to-noise ratio reaching values >2 in each dimension). The filters are tuned to a particular orientation and spatial frequency and exhibit temporal modulation. The two-dimensional nonlinearities reveal complex interactions between the pairs of filters. This cell was classified as simple according to the *F1/F0* = 1.47 ± 0.21 ratio derived from its responses to moving gratings (Skottun et al. 1991) *(F*1 is the amplitude of response at the frequency of the grating and *F*0 denotes the mean evoked firing rate). The MID analysis was based on 11 463 spikes elicited by 49 152 frames presented at 33 Hz. When applied to a novel dataset, the reconstructed 3D LN model accounted for 73 ± 3% of the information encoded in the firing rate. The corresponding values for reduced models based on just the first MID or first and second MID were 29.9 ±1.0% and 55 ±2% of information, respectively. Consistent with the properties expected for a simple cell, the first MID was associated with a rectifying nonlinearity. The second component could be classified as excitatory, because the firing rate increased with the absolute value of stimulus components along this dimension (Rust et al. 2005; Chen et al. 2007). Finally, the third MID exhibited differences in the preferred orientation at different latencies to the occurrence of a spike. This dimension was suppressive, because the firing rate decreased with the absolute value of stimulus components along it. Thus, both excitatory and suppressive dimensions could be reliably estimated not only in model neurons, as in the above, but also in real V1 cells.

**Figure 6:**
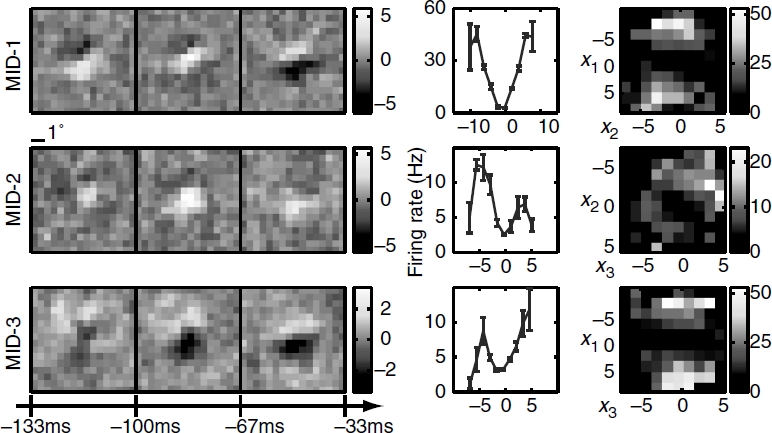
Reconstruction of an example V1 complex cell, (left) Three-dimensional spatiotemporal receptive fields for a complex cell from VI. (right) The associated one- and two-dimensional nonlinear gain functions. Neuron 946 — 2. Notations are as in Figure 1.

Complex cells are traditionally thought as presenting more of a challenge for computational methods for reconstructing neural feature selectivity. Figure 6 shows reconstruction results for three dimensions of an example complex cell. Here, we also find that all three relevant dimensions could be reconstructed reliably with peak signal-to-noise ratios >2 for each of the dimensions. The number of spikes available in this recording was 5789, which places it within the lower range of signal-to-noise values explored in Figure 4 for model neurons (the corresponding value is ≈0.4 on the *x*-axis in Figure 4). Nevertheless, when applied to a novel dataset, the reconstructed 3D LN model accounted for 72 ± 4% of the information encoded in the firing rate. We find that the relevant features of this neuron are also tuned to a particular orientation and spatial frequency, and are not space-time separable. One-dimensional cross-sections through the three-dimensional nonlinear gain function reveal that all of these three features are excitatory. This is consistent with the classical energy model (Adelson and Bergen 1997) which predicts that the first two relevant dimensions form a quadrature pair of Gabor functions with different spatial phases. The first two relevant dimensions found here are consistent with a pair of spatiotemporal Gabor functions with different temporal phases. Although all one-dimensional cross-sections of the firing rate functions are similar for the three dimensions, the two-dimensional cross-sections reveal complex interactions in how the three relevant dimensions affect the spike probability.

The analysis of these two example V1 cells illustrates the combination of dimensionality reduction and versatility that is provided by the LN model. Even with natural stimuli, it is possible to reliably estimate multiple relevant dimensions that differ in spatial and temporal phase, orientation, spatial frequency, are space-time inseparable, can be either suppressive or excitatory, and with complex input/ output functions describing the neural response in this reduced space.

**Figure 7:**
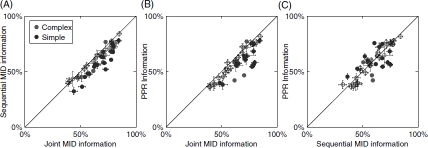
Comparison of joint and sequential methods on the responses of V1 neurons to natural stimuli. (A) Information about the responses of V1 neurons (*n* = 47) explained by three spatiotemporal dimensions estimated using either joint or sequential information maximization as a fraction of the total information per spike. Filled circles indicate cells where the joint information and sequential information were significantly different *(p<0.05)*. Overall, joint information maximization performed significantly better than sequential information maximization *(p<* 10∼^4^, paired J-test). (B) Comparison between joint information maximization and projection pursuit regression. Joint information maximization performed significantly better than projection pursuit regression *(p<* 10∼^4^, paired J-test). (C) Comparison between sequential information maximization and projection pursuit regression. The performance of the two methods was not significantly different *(p = 0.75*, paired J-test). All information values were evaluated using a set not used in estimations of the relevant dimensions.

To quantify how well the reconstructed dimensions could describe the neural responses, we computed the amount of information explained by them with respect to a novel segment of neural responses. For this purpose we used the responses to repeated natural stimuli, which also allowed us to estimate the total amount information conveyed in the firing rate. In Figure 7 we show results as a percentage of information explained by a 3D LN model for a population of 15 complex and 32 simple cells. In agreement with the analysis of model cells, we found that relevant dimensions that were estimated with the joint search explained significantly more information about the responses of V1 neurons than those estimated with the sequential information maximization (*p* = 3 × 10^−10^, paired *t*-test, Figure 7A). The same comparison held true when dimensions were estimated using PPR (*p* = 4 × 10^−8^, paired *t*-test, Figure 7B). Finally, the sequential optimization did not produce significantly different results compared to PPR (*p* = 0.75, paired *t*-test, Figure 7C). Thus, analysis of the predictive power across the population of V1 cells is consistent with the conclusion that the reconstruction of relevant dimensions from neural responses to natural stimuli requires their joint optimization.

## Discussion

Searching for stimulus dimensions that jointly account for the largest amount of the mutual information about neural responses provides a way to find the maximum likelihood LN model for a given dataset. Because information maximization can be mapped onto maximum likelihood (Kinney et al. 2007; Kouh and Sharpee 2009), the relevant dimensions obtained by information maximization have the smallest variance possible for any unbiased method. Although such properties are very valuable in the well-sampled regime, estimation of multidimensional gain functions, which is a required step of the joint search for multiple dimensions, makes the joint search subject to the curse of dimensionality (Bellman 1961). As such, joint optimization of a large number of dimensions will become inaccurate. In this article, we demonstrated that joint optimization of up to three dimensions can be done reliably for both model cells and neurons in the primary visual cortex. We have also explored two possible methods of estimating relevant dimensions. Here, analytical arguments together with the analysis of model and real V1 neurons show that sequential strategies are generally not adequate in cases where neurons are probed with natural stimuli. This conclusion contrasts with the case of uncorrelated inputs, where sequential search is adequate. However, neural responses to Gaussian inputs (even with correlations) may also be analyzed using spike-triggered covariance method (de Ruyter van Steveninck and Bialek 1988; Schwartz et al. 2006), which does not require numerical optimization and is simpler to implement.

Comparison of the accuracy with which relevant dimensions could be estimated for model cells with one, two, and three relevant dimensions provides clues as to how different optimization strategies might extrapolate to cells with a large number of relevant dimensions. Reconstruction of the first dimension usually yielded results with only small components outside the relevant subspace (e.g. Figure 1B), which however still need to be corrected in the case of multidimensional LN cell (see discussion of Equations (10)–(12)). Sequential reconstruction of the second dimension relies on that of the first, making the systematic biases more obvious qualitatively (Figure 1), and quantitatively (Figure 4). Here, although the estimation accuracy of sequential search was substantially reduced for 2D model cells compared to 1D model cells, the accuracy did improve with increasing number of spikes, which provides better sampling of the probability distributions (Figure 4). In contrast, the subspace projection between reconstructed and model dimensions for sequential optimization on the 3D model cells did not increase with increasing number of spikes. This indicates that systematic biases of the 3D sequential search were larger than the uncertainties due to undersampling even for the smallest number of spikes. The sequential reconstruction of the third dimension relies on successful optimization of the first and second dimension. Thus, large systematic biases in the reconstruction of the 3D model suggest that during sequential optimization, systematic biases accumulate with every subsequent search for a new orthogonal dimension.

Although we have reached different conclusions regarding the relative benefits of the joint information maximization and the projection pursuit regression, our results are not inconsistent with quantitative measurements of predictive power carried out by Rapela et al. (2010). The previous study analyzed correlation coefficients between the reconstructed and measured (or model) firing rates, and reported better predictive power of MID models compared to PPR models for an example simple V1 cell and comparable performance between the two methods (with overlapping errorbars), for example complex V1 cell and a model neuron in cases where the number of input stimuli or the effective number of repetitions in the case of the model neuron were large. For small number of inputs, Rapela et al. (2010) reported better predictive power of PPR models compared to MID models. It should be noted, however, that PPR models were derived by averaging across different jackknifes whereas MID models represented only one of jackknifes. Thus, in the previous study, MID models were derived from effectively a smaller dataset compared to PPR models. In our study, we report results based on averages across all jackknifes for all methods. Therefore, our findings of better performance by joint MID models compared to sequential PPR models are not inconsistent with the previous analyses.

In contrast to systematic biases that affect sequential searches for relevant dimensions, their joint optimization is limited by uncertainties due to poor sampling of multidimensional gain functions. Thus, the total number of dimensions that can be reliably estimated in the general case, such as from neural responses to natural stimuli, will be limited. The current algorithm allows for reliable estimation of up to three dimensions. Comparing the accuracy of joint estimation for two and three dimensions, we observed that requirements on the dataset size for a given accuracy increased linearly rather than exponentially with the number of dimensions (convergence curves overlap when plotted as a function of ratio between the number of reconstructed dimensions and the number of spikes). One possible explanation for this phenomenon is that, although the possible number of bins in empirical histograms increases exponentially with the number of reconstructed dimensions, many bins are empty. It is possible that strong correlations present in natural scenes limit the growth in the number of occupied bins to a polynomial function of the number of reconstructed dimensions. Because only occupied bins contribute to the calculation of information and we also limit the calculation of the gradient to bins that are occupied and have occupied neighboring bins, the number of bins where noise can affect the estimation of relevant dimensions may increase polynomially with *K*. Finally, the derivative (Equation (10)) is weighted by the distribution of observations, which also limits the effects of poorly sampled bins.

The underlying reason why relevant stimulus dimensions need to be estimated jointly from neural responses to natural stimuli is that, in the case of correlated non-Gaussian stimuli (including natural stimuli), optimal dimensions depend on our assumptions about the form of the nonlinear gain functions. This fact has two interesting consequences for practical computations and expected signal-to-noise ratios. First, in numerical computations nonlinear gain function cannot take a truly arbitrary form. For example, the number of bins chosen to describe its shape will effectively reflect our assumptions about how smooth this function is. Empirically, we found that reducing the number of bins for representing the gain functions leads to smoother shapes of relevant dimensions (even when the number of points over which the relevant dimensions is not reduced). This is likely to be due to the fact that the gradient is obtained through linear combination of conditional spike-triggered averages (10). Each of the conditional spike-triggered averages has a smooth profile because of correlations in the natural scenes. Therefore sharp features in the gradient arise mainly from differences in the conditional spike-triggered averages, becoming more pronounced with increasing number of bins.

In this work, we gradually increased the number of bins from 6 to 11. In this way, one can first optimize large-scale features in the relevant dimensions and then follow up with finer-scale features. One can think of other ways of parameterizing the nonlinear gain function, for example using an exponential function as proposed by (Paninski 2004), a set of polynomials as done by Rapela et al. (2010), or as a sum of Gaussians. These assumptions may lead to different estimates of relevant dimensions. Thus, changing parameterization of the nonlinear gain functions can provide a complementary way to control the receptive field smoothness compared to the more established evidence-optimization techniques that directly introduce (and learn from the data) the so-called hyperparameters that control the smoothness of receptive fields (Sahani and Linden 2003). In the case of natural stimuli, it is important that the resulting parameterization would be flexible enough to capture the main features of the nonlinear gain function. This is because the systematic errors in the estimation of relevant dimensions increase with a mismatch between the true gain function and its model (Section ‘Analysis of systematic bias of projection pursuit regression'; Sharpee et al. 2004a).

The second consequence is that the signal-to-noise (or variance) in receptive field estimates is also contingent upon our assumptions about the form of nonlinear gain functions. While it is true that information maximization yields the smallest variance for any unbiased method of receptive field estimation, this statement only holds within the same class of nonlinearities. For example, Sharpee and Victor (2009) found that the variance of relevant dimensions for a general LN model was greater than the estimation variance for relevant dimensions of a two-pathway model with the gain function given by a sum of a linear half-rectifier and a full rectifier. The relevant dimensions of the general LN model were estimated by information maximization and those of the two-pathway model could be obtained with linear methods that relied on specific properties of stimuli (which were two-dimensional Hermite functions). Because of the difference in the assumptions for nonlinear gain functions, the greater variance of MIDs in that case does not contradict their property of having the smallest estimation variance for a general nonlinearity. In the case where Hermite functions were used as stimuli, the relevant dimensions of the two-pathway model could be estimated with no systematic biases. In the case of natural stimuli, however, these comparisons suggest that one has to consider a trade-off between systematic and random sources of estimation errors in relevant dimensions. More constrained forms of nonlinear gain functions carry with them the increased risk of systematic errors (if the constraints take the model away from the true gain function) but lead to smaller random estimation errors.

## Conclusions

In this article, we demonstrated that characterization of neural feature selectivity within the framework of a general multicomponent LN model from neural responses to natural stimuli requires a joint optimization of relevant dimensions. A sequential search is, in the case of natural stimuli, generally not adequate and leads to large systematic biases that often exceed the errors due to finite sampling, even in the limit of small numbers of spikes where sampling is poor and random errors are large. We found that reliable estimation of up to three relevant dimensions, each representing a spatiotemporal filter, is possible for both model and real V1 cells. Encouragingly, simulation results indicated that requirements of the dataset size scale linearly, and not exponentially, with the number of jointly estimated dimensions.

## Appendix: Properties of subspace projection

Given an arbitrary basis of model dimensions , we can describe the components of a reconstructed dimension  that fall within the relevant subspace as

where *v^i^* are contravariant coordinates, and form the elements of the Jacobian matrix. For example, given three reconstructed dimensions , and a three-dimensional model subspace, the Jacobian will be given by

The contravariant components can be computed through the relationship that  are covariant components). Therefore, the Jacobian of the transformation from model dimensions to the reconstructed dimensions (projected onto the model subspace) is given by

The determinant of this matrix gives the change in volume associated with this transformation. To obtain the volume spanned by the reconstructed dimensions within the model space, we multiply det(*J*) by  to get

(26)

The volume fraction provided in Equation (23) is obtained by dividing Equation (26) by the volume spanned by the reconstructed dimensions, .
